# Does isolated greater trochanter implication affect hip abducent strength and functions in intertrochanteric fracture?

**DOI:** 10.1186/s12891-019-2457-8

**Published:** 2019-02-14

**Authors:** Hanru Ren, Qikai Huang, Jiawen He, Yongan Wang, Lianghao Wu, Baoqing Yu, Dianying Zhang

**Affiliations:** 1 Department of Orthopaedics, Shanghai Pudong Hospital, Fudan University, Pudong Medical Center, No. 2800, Gongwei Road, Shanghai, 200120 China; 2 Department of Orthopaedics, Peking University People’s Hospital, Peking, 100044 China

**Keywords:** Greater trochanter fracture, Intertrochanteric extension, Hip abducent strength

## Abstract

**Background:**

A fracture in the isolated greater trochanter is an infrequent type of femoral intertrochanteric fracture. The gluteus medius and gluteus minimus are abducent muscle groups with attachments located on the greater trochanter. Thus, a fracture of the greater trochanter could cause avulsion injury of these attachment points and eventually affect the abducent function of the hip joint and cause chronic pain. Despite these prospects, the impact of a greater trochanter fracture on abducent strength and hip joint function have yet to be investigated.

**Methods:**

Patients who were diagnosed with an isolated greater trochanter fracture (via computed tomography scan and X-ray) and underwent conservative treatment from June 2013 to October 2016 were included in the present study. Magnetic resonance imaging (MRI) was used to verify the morbidity of recessive fractures. Patients’ Harris Hip Scores were determined at 3 months, 6 months, and 12 months and the abducent strength and range of motion of the hip joint on the injured side were analyzed and compared to those on the healthy side.

**Result:**

Among 32 patients, there were 7 individuals diagnosed with isolated greater trochanter fractures by MRI, and 25 individuals whose fractures were found to have extended into the intertrochanteric region, wherein the recessive intertrochanteric region fractures had no relationship with patients’ age, gender, or weight. After 12 months of conservative treatment, 7 patients still complained of pain in the hip joint. The average Harris Hip Score was 87.84 ± 4.83, and the abducent range of the hip joint on the injured side (42.02 ± 13.93°) was not significantly different from that of the healthy side (46.24 ± 7.93°). The abducent strength of the hip joint of the injured side was 121.32 ± 41.06 N which was significantly lower than that of healthy side (137.44 ± 42.21 N).

**Conclusion:**

Results from this investigation suggest that an isolated greater trochanter fracture attenuates the abducent strength of the hip joint, which may be related to injuries of the ligaments and muscles around the greater trochanter. The surgical skills and methods of addressing isolated greater trochanter fractures merit further investigation.

## Background

Femoral intertrochanteric fracture is clinically common in senile fractures and is treated with surgery to ensure prompt patient ambulation [1, 2]. However, an isolated greater trochanter fracture of the femur is extremely rare in intertrochanteric fractures [3]. Although a preliminary diagnosis is possible through X-ray and computed tomography (CT) examinations, isolated greater trochanter fracture is likely to prompt occult intertrochanteric fractures as indicated by recent research [4, 5], and thus magnetic resonance imaging (MRI) remains the primary method for further determining the fracture type [6]. A meta-analysis performed by Seung-Ju Kim et al. indicated that MRI documented isolated greater trochanter (GT) fractures diagnosed on initial radiographs in only 10% patients and 90% MRI examinations revealed extension of the fracture into the intertrochanteric region [7]. Also, Lee et al. recommend that all patients presenting with an isolated GT fracture indicated via plain radiographs should undergo MRI examination [8]. A conservative treatment approach is common for patients with a greater trochanter fracture. However, a lack of understanding of such fractures and the impact of the participation in early functional exercises may result in the displacement of the fracture and pain within the greater trochanter, resulting in many patients choosing to undergo surgical treatment [9].

The gluteus medius and gluteus minimus are hip abductor muscle groups that are mainly responsible for the abduction of the hip joint and have insertions located on the greater trochanter. The cause of greater trochanter fracture may be associated with bone avulsion caused by the pulling of muscle following an acute traumatic injury. However, the displacement of bone within the greater trochanter following a fracture remains unclear, and, therefore, a conservative treatment strategy is considered to be a reliable path to recovery. Despite this notion, there is still a lack of research focusing on fractures of the greater trochanter after acute traumatic injury, and therefore it remains unclear if these maladies inflict tendon injuries, which could lead to a decrease in the hip abduction.

Therefore, the purpose of this investigation was to determine whether conservative treatment is a reliable means of addressing isolated greater trochanter fractures of the femur and to assess the effect of isolated greater trochanter fractures on the range of hip joint abduction and muscle strength (functions of gluteus medius and gluteus minimus).

## Methods

This study retrospectively analyzed patients with newly diagnosed isolated greater trochanter fracture (diagnosed via X-ray and CT 3-D reconstruction) from June 2013 to October 2016. All patients were over the age of 60 years old, were followed up for more than 1 year, and received conservative treatment following the initial fracture. Patients with intertrochanteric fractures with an obvious extension of the fracture line into the trochanteric region on the plain radiographs, a displaced greater trochanter fracture, additional fractures or injuries, contralateral hip dysplasia or difficulty walking, and those with preexisting severe internal medical disorders before the injury, such as tumors or Parkinson’s disease were excluded from the investigation.

The fractures of all included patients were non-displaced and were treated conservatively, and all underwent an MRI 10 days after admission to determine the type and range of the fracture. Patients with isolated greater trochanter fractures, confirmed by MRI, were restricted to bedrest for 1 month, with functional exercise allowed in bed. Two months later, they were able to walk without bearing any weight on the injured leg. Three months later, they gradually began rehabilitation exercises. When an MRI showed the fracture line extended to the trochanter, patients were required to stay in bed 1.5 to 2 months and were later allowed to perform functional exercises.

Patients were then followed up at 3 months, 6 months, and 12 months after fracture to evaluate the abduction strength and range of the affected and contralateral limbs as well as the patient’s hip Harris score (HHS) and any associated complications.

### Assessment of abduction strength

A hand-held dynamometer (HHD, Lafayette Manual Muscle Test System model 01165; Lafayette Instrument Company) was used to measure the maximum isometric strength of hip-joint abduction. The patient was instructed to lay on their side on the test-bed with the lateral hip, and knee crooked slightly. The tester was located 5 cm above the measured limb, and the patient was required to stretch out the limb of one side to measure its strength. The patient was then required to maintain the maximum isometric strength for more than 5 s and to repeat the exercise 2 times at 30-s intervals. The results were recorded [10].

### Assessment of abduction range

The patient was placed in a prostrate position on the test-bed. The angle data 1 between the bilateral iliac anterior spine connection and the longitudinal axis of the femoral shaft was measured and recorded with the limb in a neutral position. The patient was then instructed to extend the limb outward, and angle data 2 was measured between the longitudinal axis of the femur and the bilateral iliac anterior spine connection. The hip abduction angle was calculated by subtracting data 1 from data 2. This process was repeated 2 times at 30-s intervals.

Sample size estimation: the parameters are assumed as follows, alpha is equal to 0.05 (bilateral), 1-β is 0.75 and the ratio between the test group and the control group is 1:1, then the PASS software is used for calculation based on sample size formula of comparison between two sample means.

All statistical analyses were performed using SPSS 22.0 statistical software. Measurement data are presented as means ± standard deviation (SD). The association between the isolated greater trochanter fracture and the trochanteric fracture evidenced by the MRI were analyzed using the independent sample Student’s *t-*test. For analysis of the hip abduction, the range of motion (ROM) and abduction strength were studied using the paired *t*-test. In all tests, a *P*-value less than 0.05 was considered to be statistically significant.

## Results

**Fig. 1:**
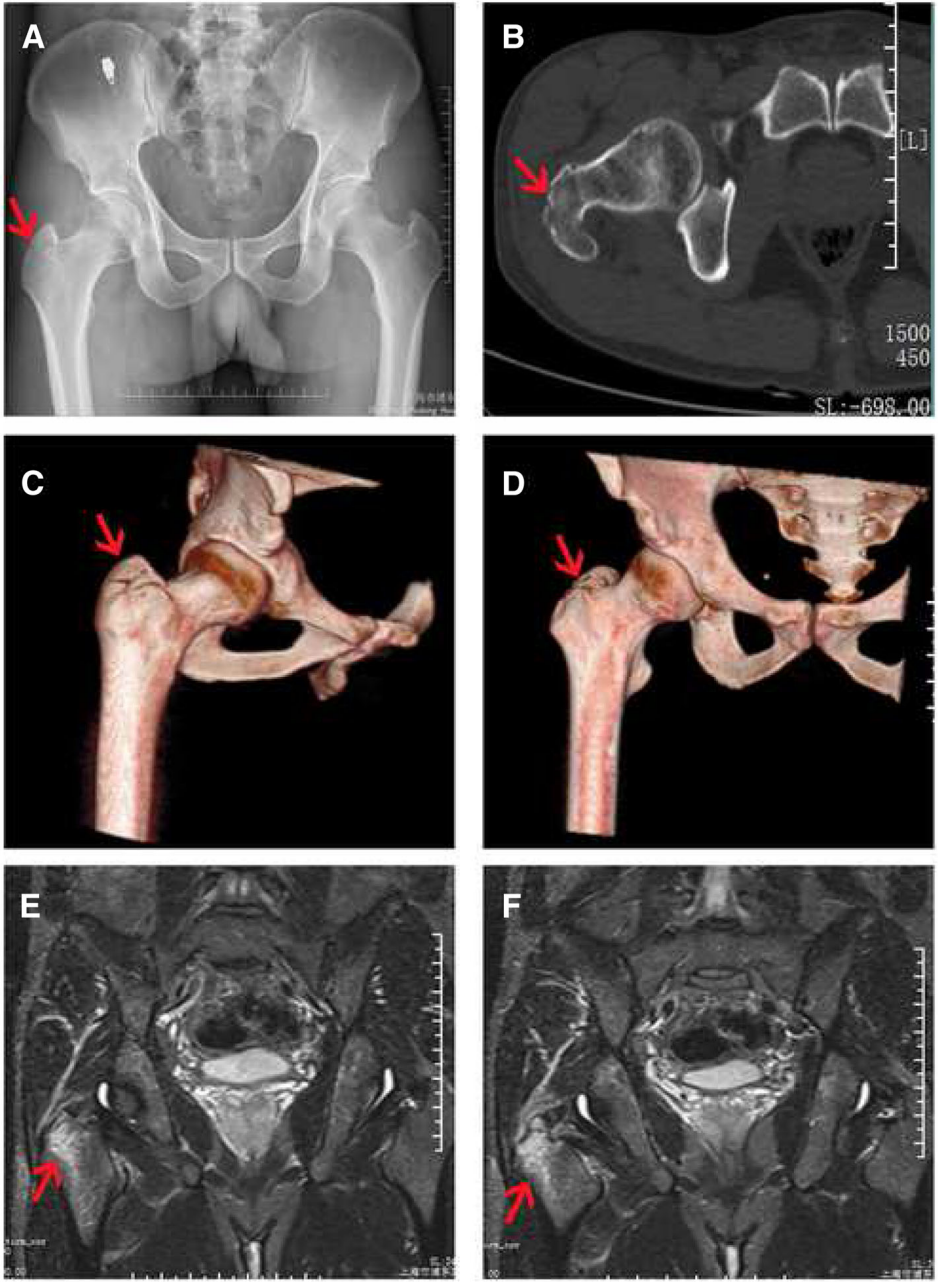
A 45-year-old man injured in a traffic accident presented with right hip pain. **a** AP radiograph showing only a minimally displaced isolated fracture of GT (red arrow). **b**, **c**, **d** CT and three-dimensional reconstruction image revealed a non-displaced greater trochanteric fracture (red arrow). **e**, **f** MRI revealed a fracture from the GT leading toward the lesser trochanter (red arrow)

A total of 37 patients were diagnosed with isolated greater trochanter fractures. One patient suffered from multiple injuries, one lost a portion of muscular strength due to cerebral infarction before the fracture, 3 patients were lost during the follow-up period, and 32 patients were ultimately confirmed the diagnosis. As confirmed via MRI, 7 patients were diagnosed with isolated greater trochanter fractures, and 25 patients were diagnosed with fractures of the trochanteric region and all the patients were found a high signal intensity of injury at the dead point of gluteus medius (a representative example is presented in Fig. 1). Nine cases were attributed to traffic accidents, 18 suffered falls, 3 suffered sprains, and one was struck during an act of violence. Patients’ age, gender, weight, length of duration of hospital stay, and follow-up time are listed in Table 1. The mean age was 72.32 ± 5.21 years, and the follow-up time was within the range of 12.36 ± 2.17 months.

**Table 1:** Demographic data and baseline characteristics

	Case(n)	N of GT Fracture on MRI	N of IT Fracture on MRI	*P*
Age (years)	72.32 ± 5.21	68.34 ± 8.16	74.74 ± 7.93	0.105
Gender (male/female)	11/21	3/4	8/17	0.201
Weight (kg)	68.07 ± 5.70 (52–82)	64.16 ± 3.14 (52–77)	71.57 ± 6.06 (60–82)	0.130
Length of stay (day)	6.20 ± 1.27 (3–9)	4.88 ± 0.79 (3–6)	7.94 ± 2.16 (5–9)	0.043*
follow-up (month)	12.36 ± 2.17	12.44 ± 1.96	12.08 ± 3.14	0.311

*GT* greater trochanter, *IT* intertrochanteric

*P < 0.05 was considered significant

The patients’ main complication was pain in the hip joint. Among the 32 patients who received conservative treatment, 7 still suffered from the pain in the hip joint one year after treatment.

After 12 months of conservative treatment, the patients’ average HHS was 87.84 ± 4.83. MRI indicated that the HHS of patients whose fracture lines did not reach the intertrochanteric region was 90.24 ± 4.07, which was numerically higher than in patients whose fracture lines did reach the intertrochanteric region (87.25 ± 4.59). However, this difference was not statistically significant. The HHS was 81.31 ± 4.21 and 71.59 ± 5.80 for patients who received 6-month and 3-month treatments, respectively. After 6 months of conservative treatment, the Visual Analogue Score (VAS) was 1.20 ± 2.12, which revealed that the pain had been significantly attenuated since the reported score of 2.64 ± 3.81 at 3 months. However, there was no statistical difference between whether the fracture line reached to the intertrochanteric part and patients’ VAS (visual analog score) (Table 2).

**Table 2:** Result of the Visual Analogue Score and Hip Harris Score for 3, 6, 12 months

	3 Months				6 Months				12 Months			
	Total	GT Fx on MRI	IT Fx on MRI	*P*	Total	GT Fx on MRI	IT Fx on MRI	*P*	Total	GT Fx on MRI	IT Fx on MRI	*P*
HHS	71.59 ± 5.80	73.58 ± 4.55	71.21 ± 5.60	0.201	81.31 ± 4.21	83.45 ± 6.13	79.59 ± 3.28	0.151	87.84 ± 4.83	90.24 ± 4.07	87.25 ± 4.59	0.320
VAS	2.64 ± 3.81	2.34 ± 2.10	2.71 ± 1.74	0.225	1.20 ± 2.12	1.22 ± 1.71	1.33 ± 1.23	0.190	1.02 ± 1.12	1.03 ± 1.22	1.00 ± 0.61	0.407

*GT* greater trochanter, *IT* intertrochanteric

*P* < 0.05 was considered significant

**Fig. 2:**
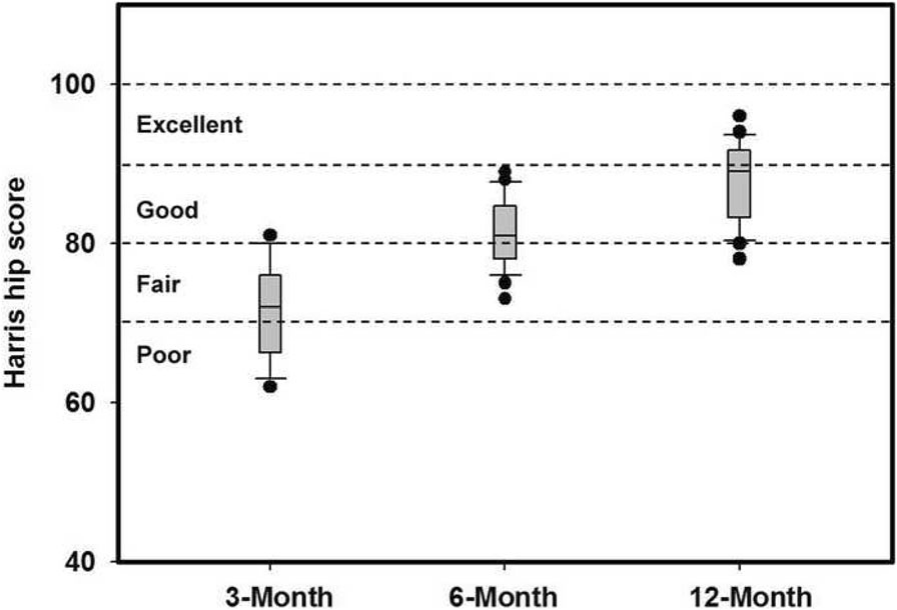
The Box-plot of Harris hip scores. Harris hip scores at 3-month, 6-month, and 12-month follow-up visits

The box-plot shows that functional outcome scores significantly improved from preoperatively to one month after fracture. The mean HHS was 71.59 (range, 62–81) at 3 months, 81.31 points (range, 73–89) at 6 months, 87.84 points (range, 78–96) at 12 months follow-up (Fig. 2).

The abducent range and strength of the hip joint were measured after the treatments occurring at 3, 6, and 12 months. After 1 year of conservative treatment, there were no significant differences between the abducent ranges of the hip joint of injured and uninjured side (injured: 46.24 ± 7.93°, uninjured: 42.02 ± 13.93°). Conversely, after 1 year of conservative treatment, relative to the uninjured side, the abducent strength of hip joints on the injured side was significantly greater (injured 137.44 ± 42.21 N, uninjured 121.32 ± 41.06 N, Table 3).

**Table 3:** The Result of the patients’ abducent range and strength

	3 Months			6 Months			12 Months		
	Healthy side	Injured side	*P*	Healthy side	Injured side	*P*	Healthy side	Injured side	*P*
Hip abduction ROM (°)	45.81 ± 9.10	23.94 ± 12.21	0.031*	46.32 ± 8.23	36.01 ± 14.13	0.085	46.24 ± 7.93	42.02 ± 13.93	0.132
maximal abduction strength(N)	126.71 ± 35.49	75.24 ± 10.68	0.014*	130.47 ± 30.59	110.59 ± 22.67	0.028*	137.44 ± 42.21	121.32 ± 41.06	0.042*

*P < 0.05 was considered significant

## Discussion

Isolated greater trochanter fractures are relatively rare among hip fractures. Fractured parts of the bone can be seen by conventional X-ray and CT scans [4, 11]. However, these imaging techniques often miss portions of the fracture, which can give rise to displacement that may necessitate a secondary operation or other complications. Current research indicates that the diagnosis of isolated greater trochanter fractures still relies heavily on MRI. According to our clinical observations, these types of injuries may be caused by acute sprains instead of falls. Patients with greater trochanter fractures are typically young. In the present investigation, among a total of 32 cases of greater trochanter fracture shown by X-ray and CT imaging, only 7 cases were isolated. This is in stark contrast to the results reported by Seung-Ju Kim et al. (only 11 cases were diagnosed by MRI to be isolated greater trochanter fractures among 110 cases), who showed a lower probability of isolated greater trochanter fracture diagnosis via X-ray and CT scanning when compared to MRI. Whether the fracture extends into the lesser trochanter is likely not associated with age, gender, weight, or other associated factors. However, these results further support the critical role of MRI in the diagnosis of greater trochanter fracture indicated by X-ray and CT [6]. In the remaining patients, the fracture lines extended into the lesser trochanter to different extents.

Surgical and conservative treatment strategies are the two principal clinical options for isolated greater trochanter fractures. The surgical method involves fixing the fracture with a dynamic hip screw [11, 12]. Lee KH et al. found patients to have fewer postoperative complications after DHS [8]. However, results from our experience suggest that surgical treatment may increase not only the patients’ pain but also their economic burden. Greater trochanter fractures do not compromise the integrity of the stress-bearing part of the greater trochanter in the thighbone and thus do not impact the stability of the pressure side during the biomechanical action. Therefore, when the fracture line extends into the lesser trochanter, the fracture remains relatively stable and can be cured by prolonged bed rest. Our study suggests that the outcomes of treatment in patients with a greater trochanter fracture are satisfactory if treatment includes stringent bed-rest immobilization and targeted rehabilitation exercises. We recommend that patients with an MRI diagnosed greater trochanter fracture be prescribed bed-rest for 1 month, with an extension to 1.5 to 2 months for those patients with fracture lines extending to the lesser trochanter. The HHS score of 32 patients’ posterior hip joint was 87.84 ± 4.83 12 months after conservative treatment. However, we also noticed sequelae after the conservative treatment for the greater trochanter fracture. Pain in the hip joint was the most common complication and was observed in 7 out of 31 patients. Results from a previous investigation suggest that the pain in the hip joint may be related to ligamentous injury [13].

Ligamentous injury in patients afflicted with a greater trochanter fracture may be unavoidable. There are numerous muscles near the dead point in the greater trochanter, which include abductors such as the gluteus medius. Avulsion in the abductor’s muscles occurs, at least in part, to tension at the time of fracture. This is the main reason for pain in the posterior hip joint following treatment. Further atrophy of the injured abductors may cause a reduced range of motion and muscle strength during the abduction. Thus, it is likely that the degree of impact on the range and muscle strength of the affected hip during abduction is dependent on the extent of the fracture. Despite this notion, there are no investigations into the changes in strength or range of abduction after treatment for patients with an isolated greater trochanter fracture. In our study, we observed a marked difference between ranges of abduction on affected and unaffected sides of the hip joint within 6 months of conservative treatment for the fracture. While, at 1 year following conservative treatment, the range of abduction on the affected side remained narrower than on the unaffected hip joint, the difference failed to reach statistical significance. The strength of abduction remained significantly lower than that of the unaffected hip. Thus, these results of support our original hypothesis.

## Conclusions

Results from the present investigation suggest that the diagnostic capabilities of X-ray and CT technology to assess isolated greater trochanter fractures are unreliable, and therefore, the final diagnosis should be made via MRI. Also, despite the persistent hip pain, our data suggest that conservative treatment supplemented with effective functional rehabilitation exercises can offer a satisfactory functional outcome for isolated greater trochanter fracture than surgery alone. Furthermore, abduction strength of the hip joint may be reduced as a result of the conservative treatment. This means that a large trochanter fracture and associated torn ligaments are more closely linked to the strength of abduction of the hip joint. Future investigations will shed light on the necessity of surgery and the efficacy of specific surgical techniques aimed at improving the functional outcome of this patient population.

## Abbreviations

GT: Greater trochanter
HHD: Hand-held dynamometer
HHS: Hip harris score
IT: Intertrochanteric
ROM: Range of motion
VAS: Visual analogue score
